# Mind bomb-1 is an essential modulator of long-term memory and synaptic plasticity via the Notch signaling pathway

**DOI:** 10.1186/1756-6606-5-40

**Published:** 2012-10-30

**Authors:** Ki-Jun Yoon, Hye-Ryeon Lee, Yong Sang Jo, Kyongman An, Sang-Yong Jung, Min-Woo Jeong, Seok-Kyu Kwon, Nam-Shik Kim, Hyun-Woo Jeong, Seo-Hee Ahn, Kyong-Tai Kim, Kyungmin Lee, Eunjoon Kim, Joung-Hun Kim, June-Seek Choi, Bong-Kiun Kaang, Young-Yun Kong

**Affiliations:** 1 Department of Biological Sciences, College of Natural Sciences, Seoul National University, San 56-1 Silim-dong Gwanak-gu, Seoul, 151-747, South Korea; 2 National Creative Research Initiative Center for Memory, Department of Biological Sciences, College of Natural Sciences, Seoul National University, Gwanangno 599, Gwanak-gu, Seoul, 151-747, South Korea; 3 Department of Brain and Cognitive Sciences, College of Natural Sciences, Seoul National University, Seoul, 151-747, South Korea; 4 Department of Psychology, Korea University, 5-1 Anam-dong Seongbuk-gu, Seoul, 136-701, South Korea; 5 Division of Molecular and Life Sciences, Pohang University of Science and Technology, Pohang, Kyungbuk, 790-784, South Korea; 6 National Creative Research Initiative Center for Synaptogenesis and Department of Biological Sciences, Korea Advanced Institute of Science and Technology, Daejeon, 305-701, South Korea; 7 Department of Anatomy, School of Medicine, Kyungpook National University, 2-101, Dongin-Dong, Daegu, 700-422, South Korea

**Keywords:** Mind bomb-1, Notch, Synaptic plasticity, Memory, Hippocampus

## Abstract

**Background:**

Notch signaling is well recognized as a key regulator of the neuronal fate during embryonic development, but its function in the adult brain is still largely unknown. Mind bomb-1 (Mib1) is an essential positive regulator in the Notch pathway, acting non-autonomously in the signal-sending cells. Therefore, genetic ablation of Mib1 in mature neuron would give valuable insight to understand the cell-to-cell interaction between neurons via Notch signaling for their proper function.

**Results:**

Here we show that the inactivation of Mib1 in mature neurons in forebrain results in impaired hippocampal dependent spatial memory and contextual fear memory. Consistently, hippocampal slices from Mib1-deficient mice show impaired late-phase, but not early-phase, long-term potentiation and long-term depression without change in basal synaptic transmission at SC-CA1 synapses.

**Conclusions:**

These data suggest that Mib1-mediated Notch signaling is essential for long-lasting synaptic plasticity and memory formation in the rodent hippocampus.

## Background

The Notch signaling pathway is a signaling module that is evolutionarily conserved from nematodes to human, which plays essential roles in pattern formation and cell fate determination through local cell-cell interactions [1]. Notch signaling is initiated by the interaction of the Notch receptors with their ligands, Deltalike (Dll) and Jagged (Jag) [2,3]. These interactions induce proteolytic cleavages of the Notch receptors, and generate a soluble intracellular domain (Nicd) that translocates to the nucleus to form a transcriptional activator complex with Su(H)/CBF1/RBP-Jκ. This complex activates the basic helix-loop-helix (bHLH) repressors, such as Hes1 and Hes5 [4]. Notch signaling is implicated in brain development by regulating cell-fate decisions and proliferation of progenitors [5,6]. In addition, Notch signaling is also involved in structural maturation of postmitotic neurons, stimulating neurite branching but inhibiting neurite growth in primary cultured neurons [7,8] and in adult-born neurons in the early stages of maturation in the dentate gyrus (DG) [9]. It has been suggested that Notch signaling plays an important role in cognitive functions, such as long-term memory and synaptic plasticity [10]. Mice heterozygous for Notch1 or RBP-Jκ display deficits in the formation of long-term spatial memory, but not in the acquisition of new information or in the formation of short-term memory [10,11]. In addition, mice overexpressing Notch1 antisense mRNA (NAS mice) showed impaired early-phase long-term potentiation (LTP) and enhanced long-term depression (LTD) at the CA3-CA1 synapses in the hippocampus [12]. In these genetic models, however, Notch signaling could have been previously altered during development as well as during functional maturation of postmitotic neurons. Moreover, it has been reported that activity-induced Notch signaling in neurons requires *Arc/Arg3.1* and is essential for synaptic plasticity in hippocampal networks [13]. However, it is still unclear whether these impaired cognitive functions are due to defective Notch signaling in mature neurons or structural changes of postmitotic neurons during development.

Mib1 regulates the endocytosis of Notch ligands to promote Notch activation in the signal-receiving cells [14-16]. Since Mib1 functions in the signal-sending cells and is required for both Deltalike- and Jagged-mediated Notch signaling in mammalian development [17], *mib1* conditional knockout mice were proved to be an excellent model to elucidate the requirement of Notch signaling in diverse processes of various tissues [18-20]. Especially, *mib1* ablation in the developing brain resulted in complete blockage of Notch signaling and the premature differentiation of radial glial cells, suggesting that Mib1 is essential for Notch signaling during embryonic neurogenesis [21].

Here we have generated conditional knockout mice of *mib1* gene in the differentiated excitatory neurons of the adult brain using *CaMKII*-*cre* transgenic mice. These *CaMKII-Cre*; *mib1*^f/f^ (*mib1* cKO) mice displayed the marked reduction of Notch signaling in the adult brain, but did not exhibit changes in neuronal morphology or structural synaptic connectivity. However, hippocampus-dependent long-term memories, such as object recognition memory, contextual fear memory, and spatial memory in Morris water maze task, were severely impaired in *mib1* cKO mice. Moreover, acute hippocampal slices from *mib1* cKO mice showed impaired late-phase LTP and LTD. Interestingly, L-LTP impairment in *mib1* cKO mice was totally recovered by expression of a constitutively active form of Notch1 (NICD). These results suggest that Mib1-mediated Notch signaling between excitatory neurons is essential for long-lasting synaptic plasticity and memory formation in the hippocampus.

## Results

### Mib1 expression in mature neurons of the adult brain

**Figure 1:**
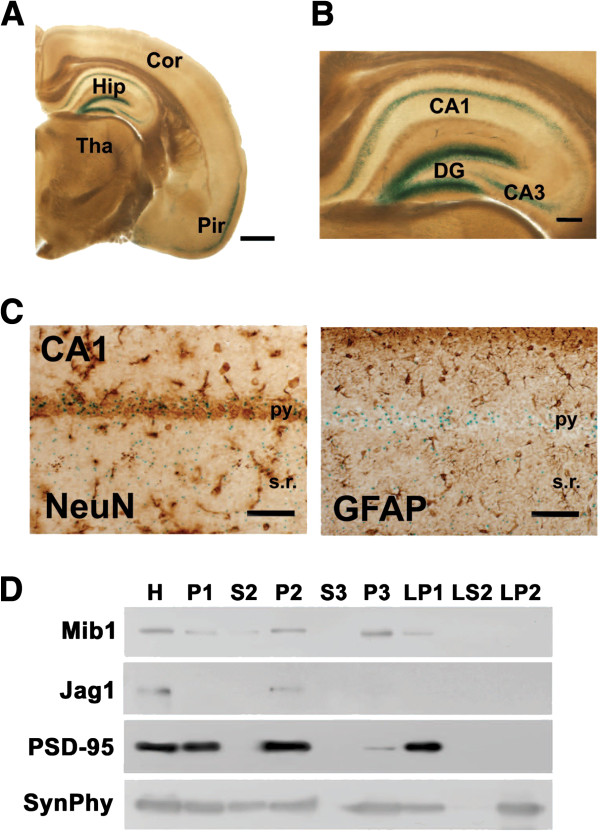
**Mib1 expression in neurons of the adult brain.** (**A**) X-gal-stained section of the adult *mib1+/LacZ* brain. X-gal reactivity was strong in the hippocampus and in the piriform cortex. Hip, hippocampus; Cor, cortex; Pir, piriform cortex; Tha, thalamus. Scale bar: 1 mm. (**B**) X-gal reactivity was high in the granule layers of the dentate gyrus (DG) and in the pyramidal layers of the CA1 and CA3 regions. Scale bar: 200 μm. (**C**) NeuN (left panel) and GFAP staining (right panel) on an X-gal-stained section of the adult *mib1+/LacZ* brain. X-gal-stained cells were merged with NeuN + neurons but not with GFAP + astrocytes in the hippocampal CA1 region. py, pyramidal neuron layer; s.r., stratum radiatum. Scale bars: 50 μm. (**D**) Distribution of Mib1 in subcellular fractions of adult rat brain. Note that Mib1 proteins were mainly detected in synaptic fractions, including P2 and LP1 and also in P3. Jagged1 (Jag1), one of candidate substrates of Mib1, was also detected in P2. PSD-95 and synaptophysin (SynPhy) were probed for comparison. H, homogenates; LP1, synaptosomal membranes; LP2, synaptic vesicle-enriched fraction; LS2, synaptosomal cytosol; P1, crude nuclear fraction; P3, light membranes; S3, cytosol.

During embryonic neurogenesis, Mib1 is expressed in intermediate progenitor cells and newborn neurons but not in radial glial cells and postmigrating neurons [21]. For detailed analysis of Mib1 expression in the adult brain, we used *mib1* knockout mice, which contain a LacZ reporter transgene in the *mib1* genomic locus [15]. X-gal staining of the *mib1*^*+/LacZ*^ forebrain revealed that β-galactosidase activity was intensively detected in the hippocampus and the piriform cortex, and was significantly detected in the cortex and the striatum (Figure 1A). In the hippocampus, granule cells in the DG most strongly expressed Mib1 and pyramidal neurons in the CA1 and CA3 region also showed high expression of Mib1 (Figure 1B). Costaining with NeuN (astrocyte marker) and GFAP (astrocyte marker) revealed abundant β-galactosidase activity in the NeuN^+^ neurons but no significant activity in GFAP^+^ astrocytes (Figure 1C), suggesting that Mib1 might function in mature neurons.

To further examine the localization of Mib1 protein, we performed subcellular fractionation of brain homogenates using differential centrifugation [22]. As a result, Mib1 proteins were mainly detected in synaptic fractions, including the crude synaptosomal (P2) and synaptic plasma membrane fractions (LP1) as well as the intracellular light membrane fraction (P3) (Figure 1D). A Notch ligand, Jagged1, was also present in the P2 fraction, suggesting that Mib1-mediated Jagged1 endocytosis [15] might occur to activate Notch signaling at synapses. Taken together, we found that Mib1 is expressed in mature neurons in the adult brain, indicating that Mib1 might have a role in neuronal function in the adult brain.

### Impaired Notch signaling in mature neurons of *mib1* cKO brains

**Figure 2:**
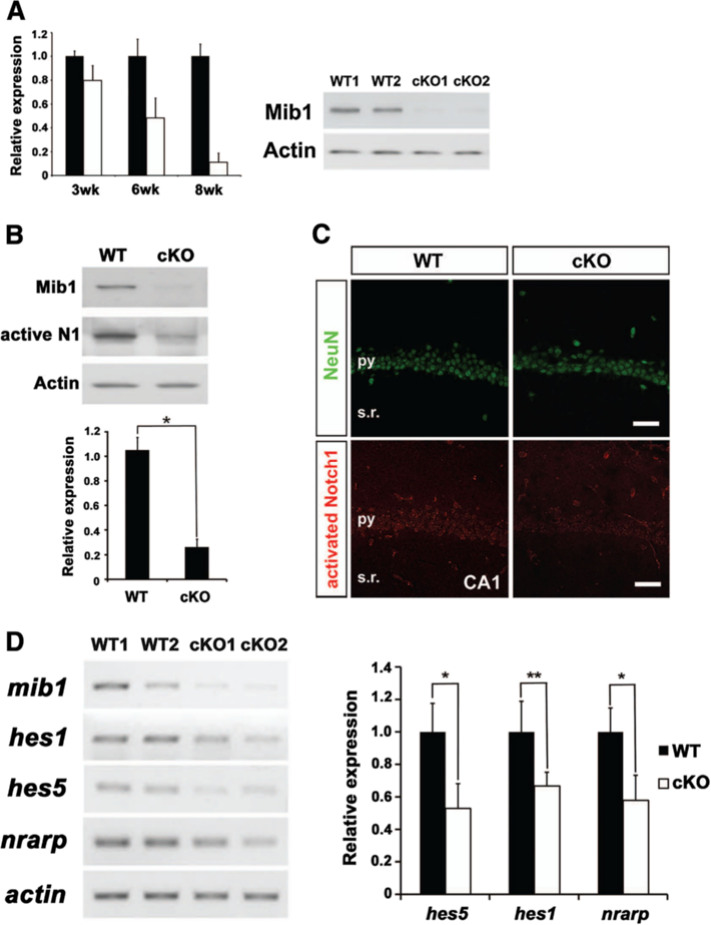
**Reduced Notch signaling in the hippocampus of *mib1 *cKO mice.** (**A**) Mib1 deletion efficiency in *mib1* cKO brains. Total RNA from 3-week-old, 6-week-old, and 8-week-old wild-type and *mib1* cKO hippocampi were analyzed by quantitative real-time PCR for *mib1* mRNA (left panel). Immunoblotting of Mib1 in the hippocampal lysates from 6-month-old wild-type and *mib1* cKO mice (right panel). (**B**) Immunoblotting of activated Notch1 in the hippocampal lysates from 6-month-old wild-type and *mib1* cKO mice. (**C**) Confocal images of NeuN and activated Notch1 coimmunoreactivity on the CA1 regions of 4-month-old wild-type and *mib1* cKO hippocampi. py, pyramidal neuron layer; s.r., stratum radiatum. Scale bars: 50 μm. (**D**) Total RNA from the 4-month-old wild-type (*n* = 4) and *mib1* cKO hippocampi (*n* = 4) were analyzed by semiquantitative RT-PCR for general Notch downstream genes, *hes1*, *hes5,* and *nrarp* (left panel). The same samples were also analyzed by quantitative RT-PCR (right panel). Error bars show standard deviation. *Significant difference; *p* < 0.001. **Significant difference; *p* < 0.02.

To ablate the *mib1* gene in the adult brain, we crossed *mib1*^f/f^ mice in which exons 2 and 3 of the *mib1* gene were flanked by loxP sites [17] with a transgenic mouse line that expressed Cre recombinase under the control of the *CaMKII* promoter [23]. It has been reported that Cre-mediated genomic recombination is restricted to postmitotic excitatory neurons in the forebrain after development [23]. As expected, genomic recombination of the *mib1* locus was achieved throughout the forebrain of adult *CaMKII-Cre*; *mib1*^f/f^ (*mib1* cKO) mice (data not shown). The *mib1* transcript and Mib1 protein levels in the hippocampus were significantly reduced in 2-month-old *mib1* cKO mice compared to wild-type mice (Figure 2A).

Previously, several studies have shown that Notch1 [9,24] and Notch ligands (Deltalike-1, Deltalike-3, Jagged-1 and Jagged-2) [24,25] are differentially expressed in differentiated neurons of the neocortex and the hippocampus. Moreover, a well-known Notch downstream effecter gene, Hes5, is expressed in the neocortex and the hippocampus [24], suggesting the presence of the active Notch signaling in the adult brain. Because Mib1 is essential for Notch signaling in the developing brain [21], it is possible that Mib1 is also indispensable for the proper Notch signal transduction in the adult brain. To examine the change in Notch signaling in the adult brain of *mib1* cKO mice, we first assessed the generation of the Notch1 intracellular domain (NICD) in the hippocampal lysates using the antibody specific to the cleaved form of NICD (activated Notch1) [26]. As a result, *mib1* cKO hippocampi showed significantly reduced NICD generation (25.02 ± 20.07% of wild type immunoreactivity) compared with the wild-type hippocampi (*p* < 0.001; Figure 2B). Moreover, immunohistochemical analysis showed decreased immunoreactivity of the cleaved Notch1 in the hippocampus and the neocortex of *mib1* cKO mice (Figure 2C and data not shown). Consistent with the decreased generation of cleaved Notch1, the expression of known Notch target genes, *hes1*, *hes5*, and *nrarp*[27], were also significantly reduced in *mib1* cKO brains compared with the wild-type brains (Figure 2D). Taken together, these results show that *mib1* cKO mice have impaired Notch signaling in the forebrain and provide an excellent loss-of-function model with which to study the role of Notch signaling in mature neurons.

### Normal brain architecture, neuronal morphology, and structural synaptic connectivity in *mib1* cKO mice

**Figure 3:**
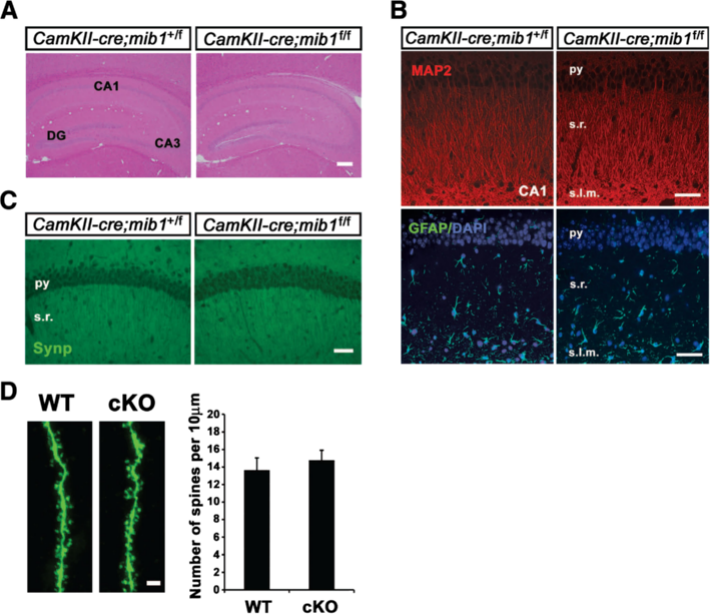
**Normal structural integrity of *mib1 *cKO brains.** (**A**) Hematoxylin & Eosin staining of paraffin-embedded sections of 6-month-old wild-type (left panel) and *mib1* cKO (right panel) hippocampi. Scale bar: 200 μm. (**B**) MAP2 (upper panels) and GFAP (lower panels) immunoreactivity in the CA1 regions of 6-month-old wild-type (left panels) and *mib1* cKO (right panels) hippocampi. py, pyramidal neuron layer; s.r., stratum radiatum; s.l.m., stratum lacunosum molecular. Scale bars: 50 μm. (**C**) Synaptophysin immunoreactivity in the CA1 regions of 6-month-old wild-type (left panel) and *mib1* cKO (right panel) hippocampi. py, pyramidal neuron layer; s.r. stratum radiatum. Scale bar: 50 μm. (**D**) Representative confocal images of neurobiotin-labeled pyramidal neurons in the CA1 regions of 3-month-old wild-type and *mib1* cKO hippocampi (left panel). Spine density in CA1 pyramidal neurons was expressed as spines per 10-μm length on secondary dendrites that were located 150–200 μm away from the cell body (WT, *n* = 9, *N* = 6; cKO, *n* = 11, *N* = 7) (right panel). The number of cells (*n*) and mice (*N*) used in each experiment is indicated. Scale bar: 2 μm. Error bars show standard deviation.

A body of evidence has demonstrated that alteration of Notch signaling in the developing brain affects neurite outgrowth and structural maturation of postmitotic neurons [7-9,28] and even the density and morphology of dendritic spines [29]. Therefore, we examined whether the integrity of brain architecture is affected in *mib1* cKO mice in which Notch signaling is impaired in postmitotic excitatory neurons. Histological analysis revealed that there was no discernible abnormality in gross brain anatomy or neuronal positioning in the forebrain of *mib1* cKO mice (Figure 3A, data not shown). In addition, the integrity of forebrains was intact in *mib1* cKO mice even at 6 months of age (data not shown). Immunohistochemical staining showed that the morphology of dendrites in the CA1 region (Figure 3B, upper panel) and in the neocortex (data not shown) of mib1 cKO mice, examined using microtubule-associated protein 2 (MAP2) immunoreactivity, was similar to that of wild-type mice. GFAP immunoreactivity revealed no astrogliosis (Figure 3B, lower panel) in the hippocampi of *mib1* cKO mice. Immunoreactivity of synaptophysin [30], a presynaptic terminal marker, in the hippocampus (Figure 3C) and in the neocortex (data not shown) was similar between *mib*1 cKO and wild-type mice. Furthermore, the number of dendritic spines was also similar between wild-type (13.69 ± 1.38 per 10 μm) and *mib1* cKO pyramidal neurons of the CA region (14.77 ± 1.11 per 10 μm, *p* > 0.2; Figure 3D). Together, these results show that *mib1* cKO mice have normal brain cytoarchitecture, neuronal morphology, and structural synaptic connectivity in our experimental condition. However, we cannot rule out a possibility that Notch signaling could affect neurite outgrowth, structural maturation, and density and morphology of dendritic spines.

### Impaired long-term memory in *mib1* cKO mice

**Figure 4:**
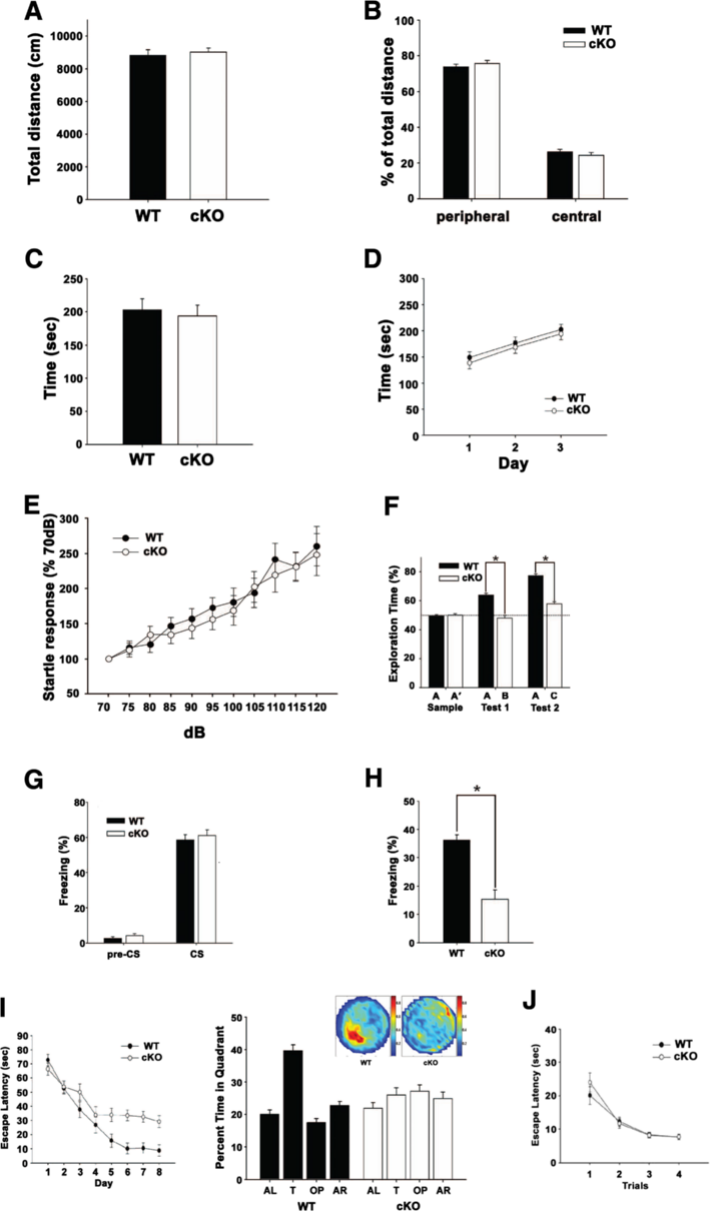
**Impaired memory in the mib1 cKO mice.** (**A**) Total path distance during the open field test. (**B**) Percentages of path distance in the peripheral region and in the central region during the open field test. (**C**, **D**) Rotarod test for motor learning. (**C**) Shows the average time spent on the rod in the fixed-speed test. (**D**) Shows the average time spent on the rod in the accelerating-speed test. (**E**) The amplitudes of the acoustic startle response for different intensities of acoustic stimuli are presented. (**F**) Mib1 cKO mice have impaired object recognition test. (**G**) Tone-dependent freezing behavior of wild-type and mib1 cKO mice at 24 h after training. The rate of freezing response was quantified before (pre-CS) and after conditioned stimuli (CS). (**H**) Freezing behavior during contextual fear conditioning test at 24 h after training. (**I**) Mib1 cKO mice showed impaired spatial memory in Morris water maze test. Mice were tested on their ability to navigate a hidden platform three times per day for 8 days. A 90-s probe trial was performed without the platform 2 h after the daily training on day 8, and staying time in each quadrant (T, target quadrant; L, left quadrant; O opposite quadrant; R, right quadrant) was recorded. Spatial histograms of the animals’ location during probe trials are illustrated. (**J**) Escape latency was normal in Mib1 cKO mice during four repetitive trials in the visible platform test in Morris water maze test. Error bars show standard error of the mean. *Significant difference; p < 0.001.

Since *mib1* cKO mice have intact brain structure integrity but impaired Notch signaling in mature hippocampal neurons, we next examined whether they show any behavioral abnormalities. In the open field task, rotarod test and startle response test, *mib1* cKO mice at 3 months of age revealed no significant alterations in general behavior and motor coordination. (Figure 4A-E).

To evaluate the consequence of Mib1 deficiency on cognitive functions, the recognition memory of 3-month-old wild-type (*n* = 22) and *mib1* cKO (*n* = 20) mice was first tested using the object recognition paradigm. In this test, both wild-type and *mib1* cKO mice spent equal amounts of time exploring two novel objects during the sample test (*t*_(40)_ = 0.15, *p* = 0.8; Figure 4F). However, when one of the familiar objects was replaced with a novel one in the first retention test, there was a significant difference in exploration time between the groups (*t*_(40)_ = 7.14, *p* < 0.001; Figure 4F). The wild-type mice spent more time exploring the novel object whereas *mib1* cKO mice failed to show such a preference. Although *mib1* cKO mice developed a slight preference for the novel object (57.8 ± 1.5%) in the second retention test, they still exhibited impairment in novel object recognition compared with wild-type mice (*t*_(40)_ = 10.3, *p* < 0.001; Figure 4F). These results show that *mib1* cKO mice have impaired recognition memory. To further examine any alterations in hippocampus-dependent forms of memory, fear conditioning was conducted with wild-type and *mib1* cKO mice. 24 h after conditioning, wild-type (58.44 ± 2.63%) and *mib1* cKO mice (61.11 ± 3.69%) showed similar levels of freezing to the CS in the cued fear memory test (*t*_(40)_ = 0.59, *p* = 0.5; Figure 4G). In the contextual fear memory test administered 24 h after conditioning, however, *mib1* cKO mice (15.39 ± 3.21%) exhibited significantly less freezing behavior than wild-type mice (36.23 ± 1.83%; *t*_(40)_ = 5.76, *p* < 0.001; Figure 4H), indicating impaired contextual fear memory in *mib1* cKO mice.

We next used the Morris water maze to investigate the effects of Mib1 deletion on hippocampus-dependent spatial memory. The wild-type mice required progressively less time to escape the platform across 8 days of training. In contrast, *mib1* cKO mice failed to exhibit improvement in finding the platform from day 5 onward (Figure 4I). An ANOVA with repeated measures revealed significant differences between the groups in escape latency (*F*_(1,40)_ = 7.36, *p* < 0.05; Figure 4I) although there was no difference in swimming speed (data not shown). During probe trials performed on day 8, *mib1* cKO mice did not show a preference for the target quadrant (*F*_(3,57)_ = 0.95, *p* = 0.4) whereas wild-type mice spent significantly more time in the target quadrant (*F*_(3,63)_ = 37.2, *p* < 0.001; Figures 4I). Furthermore, no difference was found between the groups in the visible platform test, indicating comparable motor and visual function as well as motivation between wild-type and *mib1* cKO mice (F_(1,40)_ = 0.56, p = 0.4; Figure 4J). These results show that *mib1* cKO mice have a severe deficit in spatial memory. Taken together, experiments using three independent paradigms revealed that *mib1* cKO mice have defects in hippocampus-dependent long-term memory.

### Impaired late-phase LTP and LTD in *mib1* cKO mice

**Figure 5:**
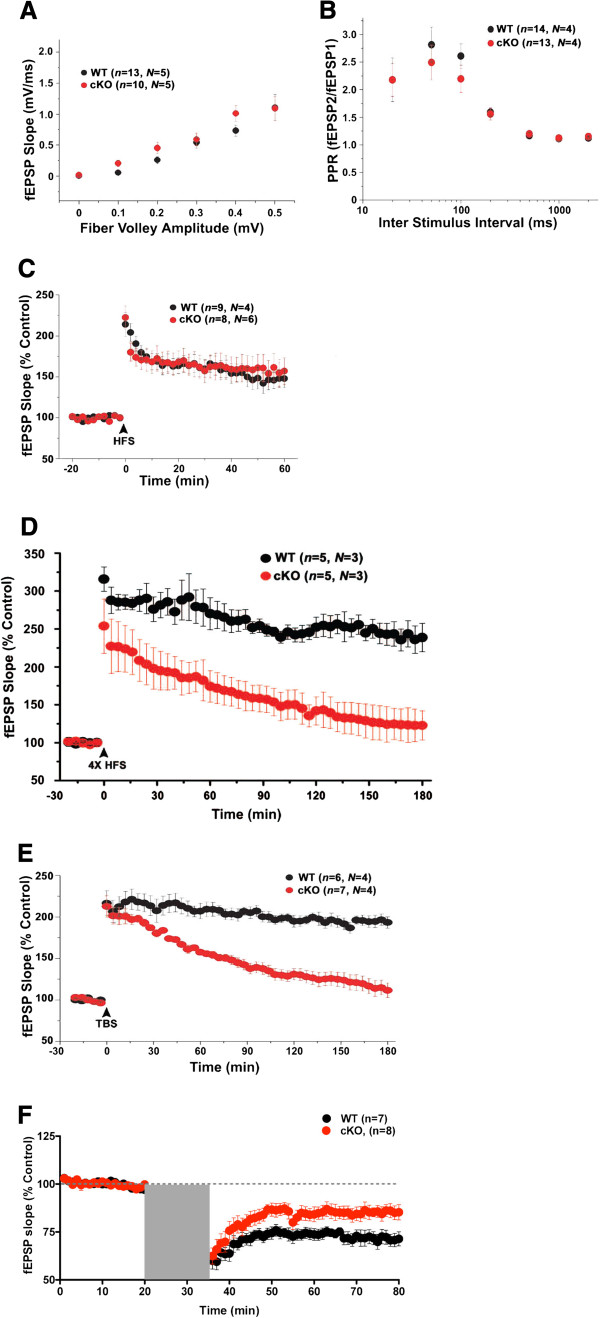
**Impaired late-phase LTP and LTD in the *mib1 *cKO mice.** (**A**) Normal synaptic transmission in *mib1* cKO mice. The synaptic input–output relationship was obtained by plotting the fiber volley amplitude against the initial slope of the evoked fEPSP. (**B**) Normal paired-pulse response ratio in *mib1* cKO mice. The graph depicts the paired-pulse response ratio (2nd fEPSP/1st fEPSP) obtained at different interstimulus intervals (in ms). (**C**) Normal E-LTP in *mib1* cKO mice. Time course of the effects of 1 train of high-frequency stimulation (HFS) on the fEPSP initial slope. (**D**) Impaired L-LTP induced by four trains of tetanic stimulation in *mib1* cKO mice. Time course of the effects of 4 X HFS stimulation on the fEPSP initial slope. (**E**) Impaired L-LTP in *mib1* cKO mice. Time course of the effects of theta-burst stimulation (TBS) on the fEPSP initial slope. (**F**) Impaired LFS-LTD in mib1 cKO mice. Time course of the effects of LFS stimulation on the fEPSP initial slope. The gray area represents the duration of LFS (low-frequency stimulation, 900 pulses at 1 Hz) for 15 minutes. The number of slices (*n*) and mice (*N*) used in each experiment is indicated in parentheses. Error bars show the s.e.m.

To examine whether mib1 cKO mice have a normal synaptic transmission or not, we performed electrophysiological analyses using extracellular field recording at SC-CA1 pathway in acute hippocampal slices. First, we found that input–output curves were essentially identical from both groups (Figure 5A), indicating that reduced levels of Notch signaling in *mib1* cKO did not affect basal synaptic transmission. In addition, paired-pulse ratio (PPR) was indistinguishable between wild-type and *mib1* cKO mice (Figure 5B), suggesting that ablation of Mib1 did not affect basal synaptic transmission at SC-CA1 synapses.

We next performed synaptic plasticity experiments, including an early-phase LTP (E-LTP) protocol induced by a single train of high-frequency stimulation (HFS), a late-phase LTP (L-LTP) protocol induced by theta-burst stimulation (TBS) and four trains of high-frequency stimulation (4× HFS) and a long-term depression (LTD) protocol induced by low-frequency stimulation (LFS). As a result, slices from both wild-type (147.83 ± 10.91%) and *mib1* cKO mice (157.06 ± 16.19%) showed significantly augmented field excitatory postsynaptic potentials (fEPSPs), with LTP lasting for at least 60 min after HFS, and no significant differences were observed between the two mouse types (*p* > 0.6; Figure 5C). However, L-LTP induction with 4 x HFS was impaired in mib1 cKO group (238.69 ± 18.57%, *p* < 0.005; Figure 5D) and the magnitude of fEPSP slope was also strongly reduced in *mib1* cKO slices (104.34 ± 7.29%) at last 5 min after TBS compared with wild-type slices (206.02 ± 14.06%, *p* < 0.0001; Figure 5E). Moreover, NMDAR-dependent long-term depression (LTD) at SC-CA1 synapses was significantly reduced in *mib1* cKO mice at last 5 min (unpaired *t*-test, p < 0.05, Figure 5F). These results show that Mib1 is required for L-LTP and LTD rather than E-LTP at hippocampal SC -CA1 synapses.

### Decreased PKMζ expression in *mib1* cKO mice

**Figure 6:**
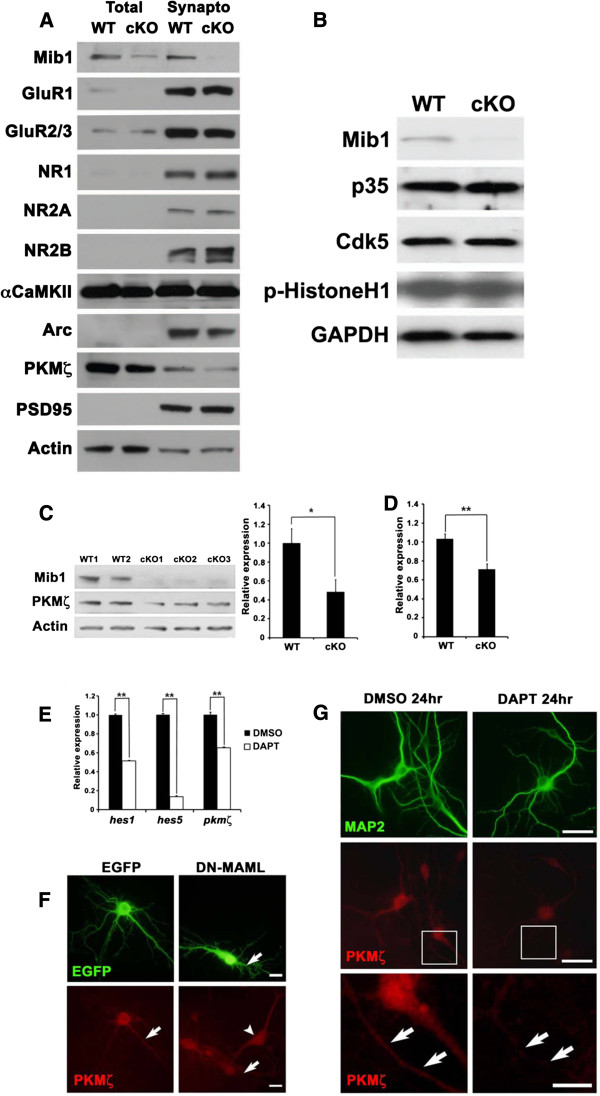
**Reduced PKMζ expression by inhibition of Notch signaling.** (**A**) Western blot analysis of glutamate receptor subunits and several proteins. Hippocampal lysates were prepared from 3 wild-type and 3 mib1 cKO mice at 6 months of age, then fractionated and subjected to immunoblotting. Note that PKMζ protein is significantly reduced in the total and synaptosomal fractions of mib1 cKO hippocampal lysate. (**B**) Hippocampal lysate from 6-month-old wild-type and mib1 cKO mice were analyzed using an in vitro Cdk5 kinase assay to determine the autoradiography using histone H1 as a substrate. (**C**) Reduced total PKMζ protein in the hippocampus of mib1 cKO mice (n = 5) compared to wild-type mice (n = 5). (**D**) Total pkmζ RNA from 6-month-old wild-type (n = 6) and mib1 cKO (n = 6) hippocampi were analyzed by quantitative RT-PCR. (**E**) Reduced pkmζ mRNA transcription by inhibition of Notch signaling in primary hippocampal neurons. DIV14 primary hippocampal neurons were treated with 20 μM DAPT for 12 h, and total RNA was analyzed by quantitative RT-PCR. (**F**) Reduced PKMζ protein expression by DAPT treatment. DIV14 primary hippocampal neurons were treated with 20 μM DAPT for 24 h and subjected to immunocytochemistry with MAP2 and PKMζ antibody. DMSO-treated cells showed robust expression of PKMζ protein in the cell body and dendrites. DAPT-treated cells showed decreased expression of PKMζ protein. Arrows in lower panels indicate PKMζ protein in dendrites. Scale bars: 50 μm in upper and middle panels; 20 μm in lower panels. (**G**) Reduced PKMζ protein expression by DN-MAML-GFP transfection. DIV10 primary hippocampal neurons were transfected with EGFP and DN-MAML-GFP DNA and subjected to immunocytochemistry on DIV14. EGFP immunoreactivity was used to identify transfected cells. EGFP transfected cells showed robust expression of PKMζ protein in the cell body and dendrites. DN-MAML-GFP transfected cells showed reduced expression of PKMζ protein, especially in dendrites. Note that nontransfected cells show significant expression of PKMζ protein. Scale bars: 20 μm. Error bars show standard deviation. *Significant difference; p < 0.01. **Significant difference; p < 0.001.

To investigate the mechanism of memory deficits and impaired synaptic plasticity in *mib1* cKO mice, we examined the expression levels of glutamate receptor subunits and several memory related proteins in total fraction and synaptosomal fraction. The expressions of glutamate receptors in synaptosomal fraction were not significantly different between wild-type and *mib1* cKO hippocampi (Figure 6A), suggesting that Notch signaling does not directly affect the expression or the stability of postsynaptic glutamate receptors. Because functional interaction between Mib1 and p35/Cdk5 was reported in the neurons [31], we also examine the expression of p35 and Cdk5 and the kinase activity of Cdk5 using hipoocampal lysates from wild-type and *mib1* cKO brains. As a result, there were no obvious differences between wild-type and *mib1* cKO brains (Figure 6B).

The atypical protein kinase C (aPKC) isoform, protein kinase M ζ (PKMζ) has been known to be critical for the maintenance of LTP and the persistence of spatial memory storage in the hippocampus [32-36]. PKMζ is responsible for the synaptic enhancement only during the late-phase LTP, but is not critical for early-phase LTP [37]. LTP induction increases new PKMζ synthesis by transcriptional regulation [38], but the upstream regulation mechanism of the PKMζ transcription has not been fully understood. Considering impaired late-phase LTP, but not early-phase LTP in *mib1* cKO mice, we postulated that defective Notch signaling might have an effect on the expression of PKMζ in *mib1* cKO mice. Indeed, the basal expression level of PKMζ was significantly reduced in the hippocampal lysate of *mib1* cKO mice at 6 months of age, as compared to that of wild-type mice (Figure 6C, *p* < 0.01). Moreover, *pkmζ* mRNA level was downregulated in the hippocampus of *mib1* cKO mice at 6 months of age (Figure 6D, *p* < 0.001), suggesting that PKMζ expression might have a role in Notch signaling. To further test the PKMζ expression by Notch signaling, γ-secretase inhibitor DAPT, which blocks Notch signaling [39], was applied to cultured primary hippocampal neurons. As expected, *pkmζ* mRNA was significantly reduced after 12 hr DAPT treatment with the decrease of Notch target genes, *hes1* and *hes5* (Figure 6E). Moreover, PKMζ immunoreactivity was also reduced after DAPT treatment and the transfection of dominant-negative Mastermind-like (DN-MAML)-GFP, which blocks the transcriptional activation of NICD [40] (Figure 6F and 6G). These results suggest that Notch signaling might be implicated in PKMζ transcription.

### Overexpression of activated Notch1 can rescue the phenotypes of *mib1* cKO mice

**Figure 7:**
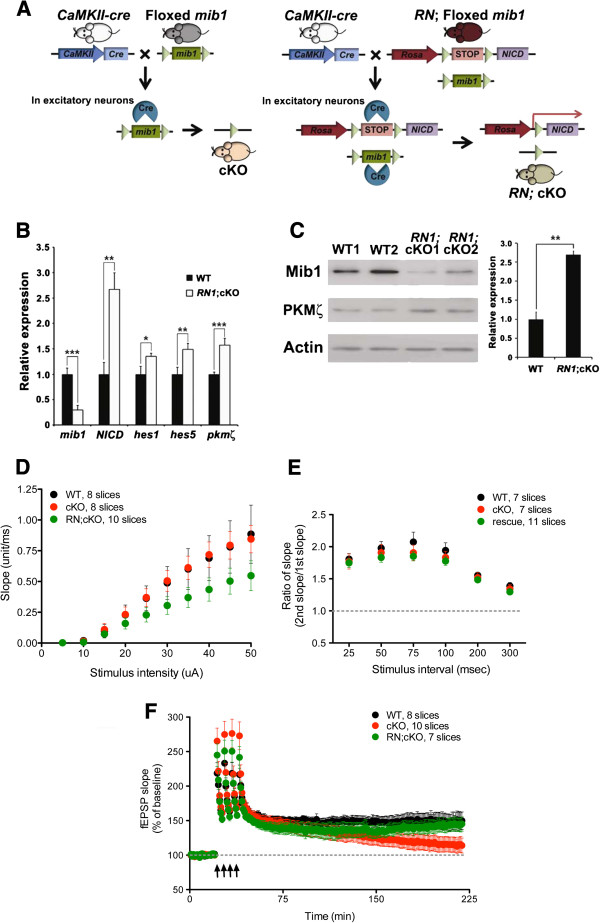
**Introduction of Notch1 ICD rescues the phenotypes of *mib1 *cKO mice.** (**A**) Schematic drawing of mouse breeding. *RN1*;*mib1* cKO mice were generated by crossing *CaMKII-cre/+;mib1f/f* mice with the *Rosa-Notch1/+; mib1f/f* mice. (**B**, **C**) Increased *pkm*ζ and Notch target genes in the *RN1*;*mib1* cKO mice. Total RNA and protein lysates from the hippocampus of 4-month-old wild-type (*n* = 4) and *RN1*;*mib1* cKO mice (*n* = 4) were analyzed by quantitative real-time PCR (left panel) (B) and by western blotting (C). PKMζ protein levels were quantified by densitometry (right panel). (**D**) Normal synaptic transmission in *mib1* cKO and RN1;*mib1* cKO mice. The synaptic input–output relationship was obtained by plotting the initial slope against the stimulus intensity (n = 8 for WT, n = 8 for cKO, n = 10 for RN1;*mib1* cKO). (**E**) Normal paired-pulse response ratio in *mib1* cKO and RN;*mib1* cKO mice. The graph depicts the paired-pulse response ratio (2nd fEPSP/1st fEPSP) obtained at different interstimulus intervals (n = 7 for WT, n = 7 for cKO, n = 11 for RN1;*mib1* cKO). (**F**) L-LTP is totally recovered in RN;*mib1* cKO mice compared to *mib1* cKO mice (n = 8 for WT, n = 10 for cKO, n = 7 for RN1;cKO).

Since Mib1 interacts with another substrates, DAPK [41] and p35 [31], we examined whether the phenotypic changes in *mib1* cKO mice are entirely caused by the defective Notch signaling. To investigate this possibility, we bred *CaMKII-cre;mib1*^*f/f*^ mice with the *Rosa-Notch1* mice [42] to overexpress NICD, a constitutively activated form of Notch1, in Mib1-deficient excitatory neurons (Rosa^NICD/+^; *CaMKII-cre*; *mib1*^f/f^, briefly, *RN1*; cKO mice, Figure 7A). To test whether exogenous NICD is overexpressed and functionally active in the hippocampus of RN1; cKO mice, we used quantitative RT-PCR to analyze expression of *NICD*[29] and Notch downstream genes, *hes1 and hes5.* As expected, *NICD, hes1* and *hes5* transcripts were significantly increased in *RN1*; cKO mice, compared to wild-type mice (Figure 7B). Simultaneously, the expression of *pkmζ* transcript (*p* < 0.001) and PKMζ protein (*p* < 0.02) were also increased in the hippocampus of *RN1*; cKO mice compared to wild-type mice, in spite of absence of Mib1 expression (Figure 7B and 7C).

In electrophysiological experiments, impaired late-phase LTP in *mib1* cKO mice was significantly rescued in *RN1; mib1* cKO hippocampal slices and these mice showed a similar fEPSP slope at last 5 min compared to that of wild-type slices (*p* > 0.6; Figure 7F). However, there were no changes in input–output curve and paired pulse ratio between groups (Two way ANOVA, p > 0.05, Figure 7D, E). These results show that impaired synaptic plasticity observed in *mib1* cKO mice is due to the defective Notch signaling.

## Discussion

It is known that Notch signaling is important for long-term memory and synaptic plasticity in *Drosophila* and mammals [11,12,43,44]. However, the previous studies did not demonstrate clearly: (1) whether deficits in memory and synaptic plasticity were caused by disrupted Notch signaling during maturation or after maturation of the brain; (2) what types of cells send and receive Notch signaling in the adult brain. In this study, we demonstrated that ablation of Mib1 after brain development causes the deficits of both hippocampus-dependent cognitive functions and synaptic plasticity. In addition, since *CamKII-cre*–mediated gene ablation is restricted only in excitatory neurons in the forebrain [23], our data show that Notch signaling responsible for long-term memory and synaptic plasticity functions between excitatory neurons in the hippocampus.

Numerous evidences have demonstrated that Notch signaling is important for structural changes in developing neurons [7-9,28]. In addition, overexpression of active Notch1 in differentiated neurons can alter neuronal morphology and structural connectivity of pyramidal neurons in the visual cortex [29]. Consistently, inactivation of Notch1 in CA1 pyramidal neurons resulted in reduced spine density [13]. In our study, however, no structural abnormalities were observed in 3-months-old *mib1* cKO brains despite reduced Notch activity. Since Mib1 acts as an E3 ubiquitin ligase that ubiquitinates Notch ligands [45], its inactivation in CA1 pyramidal neurons does not affect the expression of Notch1 itself. Thus, inconsistency in structural abnormality between models suggests that Notch1 itself may play a role in structural integrity or cleavage-independent non-canonical Notch signaling, although high doses of exogenous Notch signaling have the ability to change structural characters of differentiated neurons.

The L-LTP requires *de novo* transcription and translation [46], while E-LTP is mediated by the potentiation of glutamate receptors response at synapses without *de novo* transcription [47]. In our study, the expression levels of each glutamate receptor subunits were not significantly altered in the synaptoneurosome of the *mib1-*deficient hippocampus (Figure 6A). Considering the role of Notch as a transcription coactivator, it is plausible that L-LTP, not E-LTP, is regulated by Notch signaling. In line with this hypothesis, we observed that only L-LTP was impaired in *mib1* cKO mice and this deficit was recovered by overexpression of activated Notch1 (Figure 7F). Moreover, in our biochemical data, PKMζ, a well known protein in hippocampal L-LTP [37], was decreased in *mib1* cKO mice compared to wild-type mice, suggesting that the PKMζ expression in the hippocampus may underlie *de novo* transcription and translation by Notch signaling.

In apparent contrast to our observations, a previous report has shown that ubiquitous transgenic expression of Notch1 antisense RNA (NAS) abolished E-LTP in the hippocampus [12]. Since Notch signaling is implicated in brain development as well as in structural maturation of postmitotic neurons, it is possible that defects of E-LTP in NAS transgenic mice might be caused by developmental or structural abnormalities. On the other hand, Alberi, *et al.* showed that inactivation of Notch1 in CA1 pyramidal neurons lead to abnormalities in both E-LTP and L-LTD without any deficits in basal synaptic transmission [13]. As mentioned above, the conditional deletion of Notch1 affected spine density. Thus, we cannot exclude the possibility that reduced spine density might influence E-LTP.

Lastly, in this study, specific deletion of Mib1 in excitatory neurons using a *CamKII-cre* transgenic line caused decreased Notch signaling in the hippocampus, which was accompanied by hippocampus-dependent memory deficits and impaired L-LTP and LTD. Considering the nonautonomous role of Mib1 in signal-sending cells for the proper transduction of Notch signaling [15,21], both signal-sending cells and signal-receiving cells of Notch signaling are excitatory neurons in the hippocampus. In addition, coexistence of Mib1 and Jagged1 proteins in the synaptosome (Figure 1D) suggests that Notch-Notch ligand interaction might occur at excitatory synapses. Careful electron microscopy analysis to identify the detailed localization of Notch receptor and ligand proteins will help in probing this hypothesis in future studies.

## Conclusions

In conclusion, Mib1 is abundantly expressed and is an essential regulator for proper Notch signaling in the adult brain. In addition, Notch signaling between differentiated excitatory neurons is important for hippocampus-dependent long-term memory and late-phase LTP and LTD. Our study provides a novel mechanism for the formation and maintenance of synaptic plasticity and long-term memory via Notch1-Mib1 signaling in mammals.

## Methods

### Mice

The floxed (f) allele of *mib1* was generated previously [17]. The *CaMKII-Cre* transgenic mice [23] were obtained from Artemis Pharmaceuticals (Cologne, Germany). The *Rosa-Notch1* mice were kind gifts from Dr. Douglas Melton (Harvard University, Cambridge, MA). *CaMKII-Cre;mib1*^f/f^ mice were generated by mating the *mib1*^f/f^ mice with *CamKII-cre;mib1*^+/f^ or *CamKII-cre;mib1*^f/f^ mice. Rosa^NICD/+^; *CamKII-cre*; *mib1*^f/f^ mice were generated by mating *CamKII-cre;mib1*^f/f^ mice with Rosa^NICD/+^; *mib1*^f/f^ mice. The mice used for this study were backcrossed at least 10 generations into the C57BL/6 N background from the original genetic background. All experiments were conducted with the approval of the Animal Care and Use Committee of Seoul National University (Approval No. 081001-3).

### Electrophysiology

The fEPSPs were recorded from transverse-sectioned acute hippocampal slices (400 um thick) from mice aged 2–3 months. Mice were anesthetized with ether just before decapitation, and the hippocampal tissues were isolated from the brain and sectioned by using the Vibratome 800-Mcllwain Tissue Chopper (Vibratome, Bannockburn, IL). Acute hippocampal slices were maintained in oxygenated (95% O2, 5% CO2) artificial cerebrospinal fluid (aCSF; 119 mM NaCl, 2.5 mM, KCl, 2 mM MgSO4, 1.25 mM NaH2PO4, 26 mM NaHCO3, 10 mM Glucose, 2.5 mM CaCl2 [pH 7.4]) at 25°C for at least 1 h. The fEPSPs were recorded in the striatum radiatum of the CA1 subfield with 3 M NaCl-filled microelectrodes (3–5 MΩ) after delivering stimulation pulses (200 μs in duration) with a bipolar concentric electrode (World Precision Instruments [WPI], Sarasota, FL) to the Schaffer Collateral (SC) afferent fiber. Test fEPSPs were evoked by a stimulation intensity that yielded one third of the maximal fEPSP responses in a aCSF bath solution containing 100 μM Picrotoxin (Tocris Bioscience, Bristol, UK), and the data were acquired with an Axopatch 200A amplifier and Digidata 1200 (Axon Instrument Inc., Foster City, CA) interface. The basal responses were collected at a frequency of 0.033 Hz for 20 min. Early-phase LTP was then induced by a single train of high-frequency stimulation (HFS, 100 Hz stimulus for 1 s), and late-phase LTP was induced by the TBS protocol (five episodes of TBS at 0.1 Hz, which were composed of 10 trains [4 pulses at 100 Hz] at 5 Hz or 4 trains of HFS at 0.1 Hz).

### Behavioral tests

Adult *mib1* cKO and wild-type mice (3-month-old littermates) were used throughout all behavioral tests and all animals were managed as previously described [48]. The same mice underwent various tests in the following order: object recognition task, water maze test, and fear conditioning. Student’s *t*-tests or repeated measures ANOVA with *post hoc* pairwise comparisons (Bonferroni *t*-test) were used to determine effects of the genotype. All data are reported as mean ± standard error of the mean.

### Open-field test

The exploratory behavior of *mib1* cKO and wild-type mice was assessed in an open-field test. On the day of the experiment, the mice were transferred to a test room dimly lit by indirect red lighting and were allowed to acclimate for at least 30 min prior to testing. The apparatus consisted of a gray rectangular box (50 × 50 × 25 cm: length × width × depth), the floor of which was illuminated to approximately 60 lux. The open field was divided into a central area (30 × 30 cm) and a peripheral area. Each mouse was placed in the center of the test box and then allowed to explore the novel environment for 10 min. Their behavior was recorded and analyzed using an automated tracking system (SmarTrack). Recording parameters included the total distance traveled, the time spent in the central and peripheral areas, and the frequency of rearing and grooming. After each test, the apparatus was cleaned with a 70% ethanol solution to remove any olfactory cues.

### Rotarod test

Motor coordination and motor learning were evaluated using two different modes of the rotarod test, a fixed speed and an accelerating speed. For the fixed-speed mode, each mouse was placed on a bar (3.8 cm diameter; IITC Life Science, Woodland Hills, CA). After a 1-min adaptation period, the bar was rotated at 10 rpm for up to 300 s. Two trials were conducted and the mean latency to fall off was recorded. On the following day, the accelerating-speed rotarod test was conducted. The bar was accelerated from 4 to 40 rpm over 5 min. Each mouse performed three trials.

### Acoustic startle response test

Acoustic startle responses were measured using a standard startle reflex system, which consisted of four ventilated, sound attenuating startle chambers (50 × 50 × 50 cm), a rack-mounted operating station, and a personal computer. In each chamber, two wideband speakers (1–16 kHz) provided the audio source for the startle stimuli and background noise (60 dB), respectively, whereas a startle sensor platform, signal transducer, and load cell amplifier served to measure the animal’s startle response. The presentation and ordering of all stimuli were controlled by LabView software (National Instruments, Austin, TX). Prior to startle testing, each mouse was acclimated to a cylindrical acrylic restrainer (5 × 10 cm) for 30 min on 3 consecutive days. On the test day, the animal was placed in the restrainer, which was attached to the sensor platform. The chamber was then sealed and the mouse was given a 5-min habituation period. Once the habituation period had elapsed, 11 different intensities of acoustic stimuli (white noise, 70–120 dB for 30 ms/stimulus, in 5 dB increments) were randomly presented with an interstimulus interval of 30 s, and the amplitude of the acoustic startle response, defined as the peak voltage that occurred during the 250-ms recording window, was recorded. The startle response to each stimulus intensity was calculated as the difference in amplitude from the response following the presentation of the 70 dB (baseline) stimulus.

### Object recognition task

The apparatus was a gray rectangular box (50 × 50 × 25 cm). Each mouse was habituated to the test box for 10 min per day for 3 consecutive days. No objects were presented during the habituation period. On the sample test day, two identical objects (A and A^′^; two pyramids, 5 × 4 × 5 cm) were located symmetrically 10 cm away from the wall and separated 30 cm from each other. Each mouse was placed in the center of the test box and allowed to explore the objects for 10 min. The animal was then returned to its cage. Two retention tests were performed 24 h and 48 h after the sample test. During the first recognition test, the mouse was placed in the test box for 5 min in the presence of one familiar (A) and one novel (B; wood block, 4 × 4 × 5 cm) object. On the next day, the second recognition test was given with object A and another novel object (C; lego block, 5 × 5 × 4 cm). The objects and box were washed with 70% ethanol solution between mice. The time spent exploring the objects was recorded, and relative time spent exploring each object was calculated by dividing by the total time spent exploring the two objects. Exploration of an object was defined as directing the nose to the object at a distance of <2 cm and touching it with the nose.

### Water Maze test

The water maze is a circular metal pool (100 cm in diameter, 40 cm in height) that was filled with water (27 ± 1°C) made opaque by adding powdered milk. Detailed training procedures were provided in a previous study [49]. Briefly, each mouse was habituated to the water maze for 90 s on two consecutive days. For acquisition of spatial memory, a hidden platform (10 cm in diameter) was placed in one of the quadrants, and three trials per day were given over a period of 8 days. If the mouse did not find the hidden platform within 90 s, the animal was guided by an experimenter. After a period of 30 s on the platform, the next trial was begun. To evaluate the retrieval of spatial memory, a 90-s probe trial was performed in the absence of the platform 2 h after the daily training on day 8. The swimming path of the mice was monitored by an overhead video camera connected to a personal computer and analyzed by a tracking system (SmarTrack; Smartech, Madison, WI). The same mice were further tested in the visible platform task, which had been modified to incorporate a black plastic ball (4 cm in diameter, 7 cm high) was added to the raised platform above the water level. Four trials were given with a 40-min intertrial interval (ITI), and the location of the cued platform was moved to a different quadrant between trials.

### Contextual and cued fear conditioning tests

A Plexiglas chamber (17 × 20 × 30 cm) was used for fear conditioning. The unconditional stimulus (US) was a 0.6-mA scrambled footshock, 2 s in duration, and the conditional stimulus (CS) was an 80-dB sound at 4 KHz 30 s in duration. On the conditioning day, each mouse was located in the chamber for 3 min to measure the initial freezing level, followed by two paired presentations of the US that coterminated with the CS (ITI = 2 min). To investigate contextual fear memory, the mouse was exposed to the chamber on the next day, and the freezing response was recorded for 5 min. Subsequently, the animal was placed in a novel chamber, and the baseline freezing level was measured for 3 min before the onset of the tone. Then, the CS was presented for 3 min, and freezing behavior was analyzed (cued fear memory). Freezing was defined as no movement except for breathing.

### Immunohistochemistry

Immunohistochemistry was performed as previously described [21]. Mice were anesthetized by intraperitoneal injection with avertin and perfused with 4% paraformaldehyde (PFA) in phosphate-buffered saline (PBS). Brains were dissected, postfixed overnight at 4°C, and cryoprotected in 4% PFA/30% sucrose in PBS overnight. Next, the brains were embedded in optimal cutting temperature (OCT) compound and sectioned (14 μm in thickness) on a freezing microtome. Slices underwent antigen retrieval in 0.01 M citric acid, pH 6.0, at 100°C for 15 min.

The sections were stained with the following antibodies: mouse anti-NeuN (Chemicon, Temecula, CA), rabbit anti-cleaved-Notch1 (Cell Signaling Technology, Beverly, MA), mouse anti-MAP2 (Sigma, St. Louis, MO), rabbit anti-GFAP (Dako Cytomation, Glostrup, Denmark), and mouse anti-synaptophysin (Sigma). Alexa 488- and Alexa 594-labeled secondary antibodies (Molecular Probes, Eugene, OR) were used for secondary antibodies. For immunostaining after X-gal staining, frozen sections were soaked in X-gal staining buffer overnight at 37°C. After postfixation and washing, primary antibodies were incubated with the sections overnight at 4°C, and the secondary detection was performed using the Vectastain Elite ABC Kit (Vector Laboratories, Burlingame, CA). Images were taken using a Zeiss Axioskop 2 Plus microscope (Carl Zeiss, Göttingen, Germany).

### Neurobiotin labeling and dendritic spine counting

Hippocampal slices (400 μm in thickness) were transferred to a submerged recording chamber continuously oxygenated with aCSF. Cell bodies were visualized by infrared-differential interference contrast (IR-DIC) video microscopy using an upright microscope (Axioskop 2 FS, Carl Zeiss) equipped with a × 40/0.80 W objective (Zeiss IR-Acroplan). Negative pressure was used to obtain tight seals (2–10 GΩ) onto identified pyramidal neurons. The membrane was disrupted with additional suction to form the whole-cell configuration. Pyramidal neurons with membrane potentials below −55 mV were excluded from the analysis. Cells were held at −70 mV for about 20 min. Neurobiotin was injected through glass pipettes with 3–5 MΩ resistances containing the standard pipette solution: K–MeSO_4_, 120 mM; KCl, 20 mM; HEPES, 10 mM; EGTA, 0.2 mM; ATP (magnesium salt), 2 mM; phosphocreatine (disodium salt), 10 mM; GTP (Tris-salt), 0.3 mM; and 3 mg/mL neurobiotin (Vector Laboratories). Neurobiotin injection lasted for about 20 min. Thereafter, the patch pipette was carefully withdrawn from the membrane, and the slice was fixed with 4% PFA in PBS overnight at 4°C. After washing, nonspecific binding of antibodies was prevented by incubating the sections for 1 h with 5% goat serum in PBS and 0.3% Triton X-100. Subsequently, slices were incubated with streptavidin Alexa 488 conjugate (Molecular Probes) overnight at 4°C. Spine density on CA1 pyramidal neurons was expressed as spines per 10-μm length on secondary dendrites that were located 150–200 μm away from the cell body. All protrusions, irrespective of their morphological characteristics, were counted as spines if they were in direct continuity with the dendritic shaft. A total of 9 neurons from three wild-type animals and 11 neurons from seven cKO animals were subjected to spine density analysis. Images were taken using Olympus FV1000 confocal microscopy.

### Western blotting and Cdk5 kinase assay

For the Western blotting, the hippocampi were homogenized in lysis buffer (50 mM HEPES [pH 7.5]; 80 mM NaCl; 3 mM EDTA; 1% Triton-X 100; 1 mM dithiothreitol; 0.1 mM phenylmethylsulfonyl fluoride, 0.1 mM NaVO_4_; and 2 μg/mL each of aprotinin, leupeptin, and pepstatin). The lysates were incubated for 15 min on ice and centrifuged for 15 min at 15,000 × *g* and 4°C. The supernatant was collected as cytosolic protein extract. Generally, 10 ~ 20 μg of protein-containing supernatants were separated by size, blotted with primary and secondary antibodies, and visualized with ECL Plus (Amersham Biosciences, Uppsala, Sweden). The primary antibodies were as follows: rabbit anti-DIP-1/Mib1 (kindly provided by Dr. Patricia J. Gallagher, Indiana University, Indianapolis, IN), mouse anti-actin (MP Biomedicals, Irvine, CA), and rabbit anti-activated Notch1 (Abcam, Cambridge, MA). For the Cdk5 kinase assay, immunoprecipitated endogenous Cdk5 from hippocampi of wild-type and *mib1* cKO mice were mixed with 8 μg histone H1 peptide as a substrate in a kinase reaction buffer containing 25 mM HEPES, pH 7.4; 25 mM beta-glycerophosphate; 25 mM MgCl_2_; 100 μM Na_3_VO_4_; 500 μM DTT; and, 1 mM [γ-^32^P]ATP. The reaction was allowed to proceed at 30°C for 30 min, as described previously [50], and radioactivity was measured by autoradiography. The Cdk5 antibody (C-8) and histone H1 were purchased from Santa Cruz Biotechnology (Santa Cruz, CA) and Calbiochem (La Jolla, CA), respectively.

### Quantitative real-time PCR

For the quantitative real-time PCR, total RNA was extracted from isolated forebrains using an RNeasy Micro Kit (Qiagen, Hilden, Germany) according to the manufacturer’s instructions. Aliquots of 1 or 2 μg of RNA were used for the RT (Omniscript RT, Qiagen) with oligo-dT priming. Real-time PCR reactions were set up with each cDNA preparation in an Applied Biosystems 7300 real-time PCR system (Applied Biosystems, Carlsbad, CA) using a master mix of SYBR green I premix ExTaq (Takara Bio, Shiga, Japan) according to the manufacturer’s instructions. The levels of mRNA expression were normalized to that of β-actin. The sequences of the synthesized oligonucleotides are as follows:

*mib1*-forward: 5^′^-CCTACGACCTGCGTATCCTG-3^′^

*mib1*-reverse: 5^′^-ACCTTTCCTCTACGCCCATT-3^′^

*nrarp*-forward: 5^′^-TTTGCCACGATTAAATGTCA-3^′^

*nrarp*-reverse: 5^′^-GGGTACACAACAGCCTTCAC-3^′^

*hes1*-forward: 5^′^-ACACCGGACAAACCAAAGAC-3^′^

*hes1*-reverse: 5^′^-GTCACCTCGTTCATGCACTC-3^′^

*hes5*-forward: 5^′^- TACCTGAAACACAGCAAAGC-3^′^

*hes5*-reverse: 5^′^- GCTGGAGTGGTAAGCAG-3^′^

*NICD-*forward: 5^′^-CGTACTCCGTTACATGCAGCA-3^′^

*NICD-*reverse: 5^′^- AGGATCAGTGGAGTTGTGCCA-3^′^

*actin*-forward: 5^′^-AAGGAAGGCTGGAAAAGAGC-3^′^

*actin*-reverse: 5^′^-AAATCGTGCGTGACATCAAA-3^′^

## Competing interests

The authors declare that they have no competing financial interests.

## Authors' contributions

KJY and YYK conceived and designed the experiments. HRL and KA performed the electrophysiological analysis. YSJ performed the behavioral analysis. SYJ performed the neurobiotin labeling. MWJ performed the Cdk5 kinase assay. SKK, NSK and HWJ provided essential reagents and helped data analysis. HRL, SHA and KL performed unpulished behavioral analysis. KTK, EK, JHK JSC BKK and YYK supervised and coordinated the works. KJY, HRL, BKK and YYK wrote the manuscript. All authors read and approved the final manuscript.
